# Grain Quality, Provitamin A Carotenoid Profiles, and Sensory Quality of Provitamin A-Biofortified Maize Stiff Porridges

**DOI:** 10.3390/foods9121909

**Published:** 2020-12-21

**Authors:** Daniso Beswa, Muthulisi Siwela, Eric O. Amonsou, Unathi Kolanisi

**Affiliations:** 1 School of Agricultural, Earth and Environmental Sciences, University of KwaZulu-Natal, Private Bag X 01, Scottville, Pietermaritzburg 3209, South Africa; beswad@uj.ac.za; 2 Department of Biotechnology and Food Technology, University of Johannesburg, P.O. Box 17011, Doornfontein, Johannesburg 2028, South Africa; 3 Department of Biotechnology and Food Technology, Durban University of Technology, P.O. Box 1334, Durban 4000, South Africa; eamonsou@dut.ac.za; 4 Department of Consumer Sciences, University of Zululand, Private Bag X1001, KwaDlangezwa 3882, South Africa; KolanisiU@unizulu.ac.za

**Keywords:** biofortified maize, provitamin A retention, stiff porridge, sensory quality

## Abstract

Provitamin A-biofortified maize could contribute to the alleviation of vitamin A deficiency (VAD), which is prevalent in sub-Saharan Africa due to a high consumption of starchy, maize-based diets. Four varieties of provitamin A biofortified maize were studied for grain colour, grain texture, thousand kernel weight, and hectolitre mass. Provitamin A biofortified maize stiff porridges were prepared and their retained provitamin A was determined using lutein, zeaxanthin, β-cryptoxanthin, and β-carotene (all-trans and cis isomers) as standards. Provitamin A concentration in the biofortified porridges ranged from 2.24 to 3.18 µg/g and retention from 91–105%. Descriptive sensory analysis and the 5-point facial hedonic test were used to evaluate the sensory quality of the porridges. The biofortified maize porridges were described as sticky, fine, with high intensity residual grain, and having a slightly bitter aftertaste with a cooked maize flavour and aroma, whereas the intensities of these attributes were insignificant in white maize porridge. About 33% of the consumer sample (N = 60) liked the porridges and 28% disliked the porridges, whilst approximately 38% of the consumers were neutral. The findings suggest that biofortified maize stiff porridge can deliver a significant amount of provitamin A to targeted consumers, but the acceptance of biofortified maize still needs to be improved on.

## 1. Introduction

Maize (*Zea mays* L.) porridge is an energy-dense staple food, which dominates the daily diets of many households in most sub-Saharan African countries. It is prepared according to different consistencies, with thin/soft or stiff forms being the most popular in this region. In the thin/soft consistency, maize porridge serves as a breakfast and refreshment beverage for adults, and children consume it as a complementary food [1,2]. Stiff porridge is largely consumed as the main dish during lunch and dinner [3]. Maize porridge is often consumed with milk or meat and vegetables, but among low income consumers, the soft and stiff porridges are consumed mainly with indigenous vegetables because these consumers cannot afford the high cost of animal products. Unfortunately, the main ingredient for these porridges is white maize meal, which is known to be deficient in vitamin A. Large populations in sub-Saharan Africa are at a high risk of vitamin A deficiency (VAD), because their diets are comprised mainly of white maize and other vitamin A deficient starchy staples, with little dietary diversity [4]. As a result, maize is one of the crops being targeted for improved provitamin A carotenoids content [5,6].

Improving the provitamin A carotenoid content of staple crops through biofortification is regarded as a sustainable strategy for alleviating VAD [7]. Unfortunately, consumer studies in a number of Southern African countries have found low acceptance of the biofortified maize compared to white maize, for example in Kenya [8], Zimbabwe [9], and South Africa [10]. The biofortification process of maize increases the concentration of carotenoid pigments in the grain (both provitamin A and non-provitamin A carotenoid pigments), resulting in the grain changing colour from white to yellow (or orange) [11]. This yellow colour has significantly contributed to the poor acceptance of yellow maize, due to the fact that African consumers are accustomed to white maize, as stated earlier. In addition, carotenoids also impart other sensory properties in the yellow maize, such as an unusual flavour and aroma, making it significantly different to the white maize and further contributing to the lower acceptance of the yellow maize [9,10].

Despite the altered sensory properties, the poor acceptance of yellow maize by African consumers also seems to be caused by other factors such as demographics, psychological, and socio-economic factors. Yellow maize is negatively associated with its common use as an animal feed, and as a food aid item [9,12]. Pillay et al. [10] found that among South African consumers surveyed, younger school children preferred yellow maize, whilst older school children and adults preferred white maize over yellow maize. In Kenya, it was found that consumers with a high education level preferred white maize [8], urban consumers also preferred white maize, whilst there was a preference for yellow maize in some non-urban parts of the country [8,12]. The Kenyan consumers were found to be interested in commercially fortified maize and would buy yellow maize only if it was sold at a discounted price. In contrast, yellow maize was more acceptable in Mozambique than local white maize varieties [13]. Furthermore, the attitude of consumers towards biofortified crops was observed to change when they were educated about the nutritional benefits involved [14].

The type of food in which provitamin A-biofortified maize is presented to the consumer was found to have an influence on its acceptance [10]. In a study conducted by Pillay et al. [10] in the province of KwaZulu-Natal, South Africa, it was demonstrated that biofortified maize was more acceptable to secondary school children in the form of samp, compared to white maize samp, which was preferred by adults. This finding suggests that the acceptance of biofortified maize can be significantly higher if it is presented to the consumer in a maize food type that is yet to be established. The study of Pillay et al. [10] did not include maize stiff porridge in the consumer acceptance test; yet maize stiff porridge is arguably the most popular maize food in sub-Saharan Africa, especially in Southern Africa. Further studies are needed to evaluate consumer acceptance of stiff porridge, particularly among Southern African consumers. 

Although several studies have been conducted on consumer acceptance of provitamin A-biofortified maize, it appears that the descriptive sensory properties of the biofortified maize products have not been studied. Information about the sensory properties of provitamin A-biofortified maize may be useful in an effort to breed crops for improved product acceptability. Therefore, in this study, the sensory properties and consumer acceptability of stiff porridge made with provitamin A-biofortified maize were investigated. The other objectives of this study were to determine the quality (mainly milling quality) of provitamin A-biofortified maize grain, and the retention of provitamin A in the biofortified maize stiff porridge.

## 2. Materials and Methods 

### 2.1. Maize Grain Varieties

Four varieties of provitamin A-biofortified maize, PVAH 79–100, PVAH 1–26, PVAH 27–49, and PVAH 50–75, produced by conventional breeding methods at Cedara Research Station, KwaZulu-Natal, South Africa, were used in this study. Experimental F1 maize hybrids were developed by cross-pollination of the inbred lines where lines with deep orange colour were advanced to the next generation. According to the breeders, deep orange colour hybrids are correlated with a higher concentration of β-carotene. The hybrids were divided into groups based on colour, with each group used to make a synthetic population by mixing grain of the hybrids and allowing them to mate randomly. The synthetic population used in this study was designated PVAH. The hybrids with sufficient seeds were planted at the University of KwaZulu-Natal (UKZN) Ukulinga Research Farm, Pietermaritzburg, South Africa. Standard cultural practices for maize production were followed. A representative white maize grain was also produced under the same conditions and location as the biofortified varieties. The grain ears were harvested manually and then allowed to dry for 21 days at ambient temperature (±25 °C). After manual threshing, the grains were stored at 4 °C until used.

### 2.2. Physical Properties of Maize Grain

#### 2.2.1. Colour of Maize Grain

The colours of the provitamin A-biofortified maize grains and the representative white maize grain, were measured with a spectrophotometer (Colorflex, Hunter Associate Laboratories, Reston, VA, USA). The spectrophotometer was calibrated according to manufacturer’s instructions using white and black standardising tiles. About 20 g of maize grain was weighed into a glass sample jar, placed into the sample port and measured. The colours of three replicate grain samples of each of the biofortified varieties, in addition to the representative white grain sample, were measured and expressed using CIELAB parameters (L*, a*, b*). L* (100 = white; 0 = black) is an indication of lightness; a* measures chromaticity, with positive values indicating redness and negative values indicating greenness; and b* measures chromaticity, with positive values indicating yellowness and negative values indicating blueness [15].

#### 2.2.2. Grain Texture

The milling index was measured with the NIR method, using the Infratec 1241 Grain Analyser. The manufacturer’s recommended operating procedure was followed (Foss Tecator AB, Höganäs, Sweden). Approximately 500 g of sound kernels were placed in a hopper and analyzed according to the manufacturer’s operating procedure. The hardness of whole kernels was measured at 860 nm.

#### 2.2.3. Thousand Kernel Weight

The weight of 1000 kernels was measured to determine the kernel size, and for calculation of the sowing rate [16]. A representative sample of clean, sound, and unbroken kernels of each variety were randomly sampled and counted using a numigral seed counter (Chopin SA, Villeneuve-La-Garenne, France). The weight of 1000 kernels was measured to an accuracy of ±0.01 g and expressed as grams per 1000 kernels. The measurements were replicated three times and the average was recorded as the final result.

#### 2.2.4. Hectolitre Mass

The hectolitre mass of the grains was determined following the American Association of Cereal Chemists (AACC) Method 55–10 [17]. The hectolitre mass of grain was measured using an apparatus that consisted of a hopper and a 0.5 L receiver (cup). The grain sample was poured into the funnel, the funnel was positioned on top of the 0.5 L receiver so that notched legs of the funnel fitted firmly onto the receiver’s rim. The slide of the Cox funnel was quickly removed to allow the grain to drop evenly into the 0.5 L receiver. The funnel was carefully removed and the striker was used to scalp off the overflowing grain in the receiver. The grain was transferred into the scale pan and weighed on a standard laboratory scale. The grain weight was in kilogrammes per hectolitre (kg/hL).

### 2.3. Grain Milling

**Figure 1:**
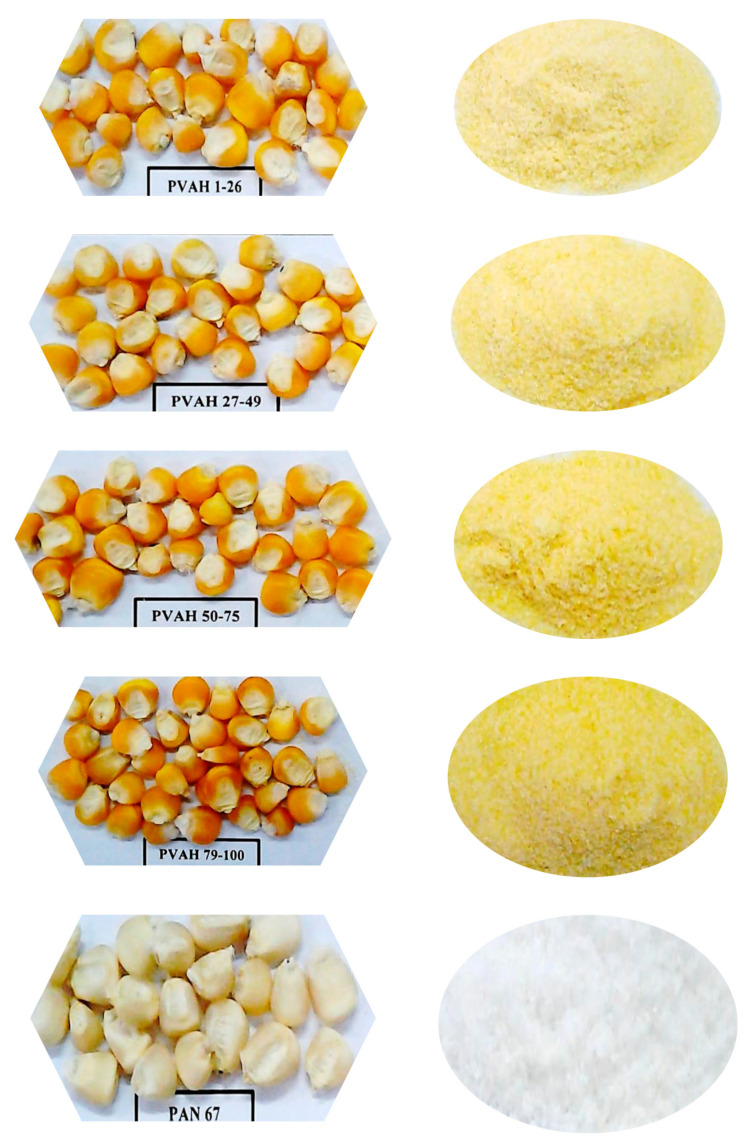
Provitamin A biofortified maize grain and their maize meal. PVAH = provitamin A hybrid; PAN67-representative white maize.

Four provitamin A biofortified maize varieties and a representative white maize variety were cleaned using a standard method. The cleaned grain was milled with a pilot plant roller mill (Model MK 150, Roff Industries, Kroonstad, South Africa) with a three-break system, which yields super meal, maize grits, and fine meal. The three-break system consisted of a set of three roller mills of decreasing roller gap size, which progressively broke up maize grain into smaller particles [18]. Super maize meal, the product that passed through the 459 µm aperture screen, was collected from the last two-break systems and used for making porridge samples. Figure 1 shows provitamin A biofortified maize grain, the representative white grain, and their respective maize meal.

### 2.4. Preparation of Stiff Porridges

Stiff porridges were made from the super meal of each of the four provitamin A-biofortified maize varieties and the reference white variety. The recipe of a typical traditional maize stiff porridge consumed by the *Venda* tribe of South Africa was standardised with the help of three experienced *Venda* women who worked at the University of Venda experimental farm, South Africa. The final maize stiff porridge recipe consisted of 1 part of super maize meal and 5 parts of water. The porridge was processed at boiling temperature (96 °C) for 65 min with continuous mixing using a whisk and wooden spoon, respectively.

### 2.5. Provitamin A Analysis and Retention 

The provitamin A content from the grains of each of the four provitamin A-biofortified maize varieties, as well as those of their porridges, was determined. Samples of maize stiff porridges prepared as described earlier were cooled, freeze-dried, and milled separately into flour using a laboratory hammer mill (Glen Creston, Stanmore, UK) fitted with a 0.5 mm aperture screen. Grain samples of each of the biofortified maize varieties were milled in the same manner as the freeze-dried porridge samples. Carotenoids were then extracted from the flours of the porridges and grains using the standard method [19,20,21]. The carotenoid extracts were analysed on a C8 column (1.7 µm; 2.1 mm × 100 mm) in an Acquity UPLC separation system (Waters Co., Milford, MA, USA) consisting of a binary Solvent Manager, Sample Manager, and PDA detector. Solvent A consisted of ammonium acetate 10 mM: 2-propanol (90:10), and Solvent B consisted of acetonitrile: 2-propanol (90:10). The flow rate was set at 0.3 mL/min, injection volume at 2 μL and absorbance measured at 450 nm. Solutions of pure carotenoid pigments that were selected on the basis that they have been found in provitamin A-biofortified maize grain were used as standards (CaroteNature GmbH), i.e., lutein [(Xanthophyll, (3*R*,3′*R*,6′*R*)-β,ε-Carotene-3,3′-diol) HPLC 94%, isolated, cryst. No. 0133], zeaxanthin [(3*R*,3′*R*)-β,β-Carotene-3,3′-diol) HPLC 97%, synth., cryst. No. 0119], β-cryptoxanthin [((3*R*)-β,β-Caroten-3-ol) HPLC 97%, synth., cryst. No. 0055], and β-carotene (all-trans and cis isomers) [(β,β-Carotene) HPLC 96%, synth., cryst. No. 0003]. The total provitamin A concentration was calculated as β-carotene using the formula [22]:

Total provitamin A = (all-trans + 9-*cis* + 13-*cis β-carotene* isomers) + 0.5(β-cryptoxanthin). (1)

The apparent retention of provitamin A in maize stiff porridge samples was calculated as follows [18]:

(2) $$\%apparent\;retention=\frac{provitamin\;A\;content\;per\;g\;porridge\;\left(dry\;basis\right)}{provitamin\;A\;content\;per\;g\;super\;maize\;meal\;\left(dry\;basis\right)}\times 100.$$

### 2.6. Sensory Evaluation

Stiff porridges were separately prepared with each of the four provitamin A-biofortified maize varieties and the representative white variety and subjected to sensory evaluation. The stiff porridges were prepared as described under 2.4 Preparation of stiff porridges.

#### 2.6.1. Descriptive Sensory Analysis

A panel of 10 trained panellists, selected from an initial 15 prospective panellists, was used for the sensory profiling of the stiff porridges. The panellists were selected based on interest, availability, and sensory acuity, which were determined during their recruitment and training. All panellists were postgraduate students in agricultural disciplines at UKZN, South Africa. The panellists were regular consumers of maize foods. During the training sessions, the panel generated and defined descriptive terms for each sensory attribute (Table 1).

At least one reference for each descriptor was identified and an intensity rating scale was developed for each attribute. The references were sourced, and the panellists used them to analyse the porridge samples as part of the training process. The reliability of the panellists was assessed during the training sessions through repeated analyses. The panellists were isolated from each other in booths during the reliability tests to prevent them from influencing each other. Five prospective panellists were withdrawn as their reliability was not satisfactory. The porridge samples were randomly labelled with three-digit codes obtained from a Table of Random Numbers. The biofortified maize porridge samples and the representative white porridge sample, along with references, were presented to the 10 panellists for analysis using the protocol of Anyango et al. [23].

The porridge samples (50 g each) were served at room temperature (25 °C) to each panellist in a randomized order, which was determined from a Table of Random Permutations of Nine. The panellists were isolated from each other in booths to prevent them influencing each other, as was done during the reliability tests. The panellists used 13 sensory descriptors to describe and rate the intensity of the sensory attributes of the stiff porridges (Table 1). The panellists handled the stiff porridges using their hands, the way stiff porridge is normally consumed. Responses were written directly onto a questionnaire which was provided to each panellist. Analysis was replicated twice.

#### 2.6.2. Consumer Acceptability Test 

Sixty (60) regular consumers of maize stiff porridge were recruited from *Ngulumbi* village in Sibasa, Limpopo province, South Africa. The consumer panel consisted of 18 males and 42 females with no allergies to maize. The four samples of biofortified maize stiff porridge, and a representative porridge sample made with white maize, were presented to each panellist in a central location. The panellists were seated far apart to prevent them from influencing each other. Sample labelling and presentation order were randomized as described under the section on descriptive sensory analysis. 

The consumer panel rated the sensory acceptability of the porridge samples (50 g each at 25 °C) in terms of colour, texture, taste, aroma, and overall acceptability using a 5-point facial Hedonic scale (in *TshiVenda* vernacular), whereby ‘5′ represented the highest possible score (like extremely) and ‘1′ the lowest possible score (dislike extremely). Panellists were also provided with tap water to rinse their palates before and between testing. The 5-point facial Hedonic scale was used instead of the customary 9-point Hedonic scale because the consumers surveyed were semi-illiterate. Longer hedonic scales, e.g., 7 or 9 ratings, tend to confuse subjects with lower literacy levels, while scales that are shorter than the 5-point scale tend to cause end-point avoidance [24].

#### 2.6.3. Ethical Considerations

Ethical approval to conduct this study was obtained from the Humanities and Social Sciences Ethics Committee, UKZN. Approval to perform the consumer acceptance test at *Ngulumbi* village in Sibasa, Limpopo province, South Africa, was obtained from the local chief who represented the tribal authority. Both the descriptive test and consumer panel members signed a consent form to indicate their consent to participate in the study and to confirm that they understood the purpose of the study. The panellists were informed that participation in the study was voluntary and they were free to withdraw from the study at any stage. The consent form for the descriptive test panel members included an agreement about payment for participation and that a panellist could be withdrawn from the study if his/her performance was not satisfactory and, in that case, they would be paid pro rata. In the consent form, it was also stated that personal information of the panellists would be kept confidential.

#### 2.6.4. Statistical Analysis

Data on the physical properties of the grain, sensory profiling, consumer acceptability, and provitamin A retention were analysed using the Statistical Package for the Social Sciences (SPSS) version 21.0 for Windows (SPSS IBM, New York, NY, USA). Comparison of multiple means was performed using the Fisher’s Least Significant Difference test (LSD) (*p* < 0.05). Furthermore, the descriptive sensory data was subjected to Principal Component Analysis (PCA) using Minitab version 16 (Minitab Inc, State College, PA, USA) to evaluate and identify variations between provitamin A-biofortified maize porridges based on their sensory attribute loadings. The Agglomerative Hierarchical Clustering (AHC) method was used for segmentation. Ward’s test was used for the allocation of panellists to clusters [25].

## 3. Results and Discussion

### 3.1. Grain Properties

Grains from the provitamin A-biofortified maize had an intense yellow to orange colour compared to the white maize grain as indicated by their high Hunter *a* values, which ranged from 16.6 to 18.9, compared to the Hunter *a* value of 6.3 for the white maize grain (Table 2). The intense yellow colour of the provitamin A-biofortified maize varieties may be attributed to the presence of colour pigments in the grain [10,26]. Carotenoid pigments, including carotenes and xanthophylls, have been found responsible for the yellow and orange colour of the maize grain endosperm [27]. However, the colour of the grain used in this study was less intense compared to the biofortified maize grain studied by Pillay et al. [10]. The colour of grain studied by Pillay et al. [10] ranged from 53.6–57.0 for lightness, 16.5–25.7 for redness, and 29.3–37.5 for yellowness.

The milling indices of the biofortified maize grains were quite similar and were all lower than the milling index (93.5) of the white maize grain. The milling index is positively correlated with grain hardness, which is the main physical parameter for selecting the maize for food use [28,29]. Often, maize is milled before it is processed into food products. Grain of acceptable milling quality should be fairly hard. The provitamin A-biofortified maize varieties used in this study could be of lower milling quality because they were not as hard as the white maize. However, the provitamin A-biofortified maize varieties should be more suitable to making stiff porridge than the white maize because maize porridges require grain with fairly low hardness [30].

The density of maize grains was evaluated in terms of thousand kernel weight (TKW) and hectolitre mass. PVAH1-26 had a significantly higher TKW (267.3 g) than the other biofortified varieties (206.5–234.4 g) and the white maize (257.1 g) (Table 2). The hectolitre mass values indicate that the biofortified maize grains had quite similar grain density, which was also similar to that of the white maize grain. The hectolitre mass is one of the grain physical traits which can be used to predict the end-use properties of the grain [31]. High hectolitre mass values are associated with intense hardness of grain and are an indication of good dry-milling quality. High hectolitre mass values (91.7–96.3 kg/hL) of provitamin A-biofortified maize varieties relative to white maize (88.2 kg/hL) were reported by Pillay et al. [32]. The observed hectolitre values in the current study were, in general, lower than those reported by Pillay et al. [32]. Chuck-Hernandez et al. [33] also reported a similar hectolitre mass value (76.3 kg/hL) for yellow dent maize. Grain density determines the type of milling suitable for the grain. Grain of low hectolitre mass usually contains a lower percentage of the hard endosperm and is suitable for wet-milling [34]. Both the thousand kernel weight and hectolitre mass measure grain density. Grain density is related to milling index (grain hardness) [35]. The differences in hectolitre mass among the provitamin A-biofortified maize varieties were not substantial and hence they should be of similar milling quality.

### 3.2. Provitamin A Retention

The biofortified maize grains had similar provitamin A contents; they ranged from 2.40 to 2.58 µg/g β-carotene (dry weight basis [DW]) (Table 3). As expected, provitamin A was not detected in the white maize grain. The retention of provitamin A in the biofortified maize stiff porridges was considerably high and varied across the different samples (91 to 123%). PVAH1-26 stiff porridge had the highest provitamin A retention, whilst the lowest retention was recorded for stiff porridge made with PVAH50-75. The low apparent retention of provitamin A carotenoids in the stiff porridges prepared with PVAH 50–75 was probably due to isomerisation of *trans*-β-carotene isomers to *cis*-β-carotene isomers. In this study, the β-carotene isomers measured in the stiff porridges were mainly *cis*-β-carotene (9-*cis*-β-carotene and 13-*cis*-β-carotene). The *cis*-β-carotene isomers are converted to vitamin A less efficiently than all-*trans*-β-carotene isomers [36,37,38]. The substantially high provitamin A retention in the biofortified maize porridges was similar to that reported by Pillay et al. [18] when provitamin A-biofortified maize was cooked into *uphutu* (crumbled maize porridge) and samp. It has been found that heat processing enhances the availability of vitamins and carotenoids by releasing them from the food matrix [39]. A significant increase in lycopene, β-carotene, and α-tocopherol content was observed after oven baking tomatoes at 160 °C [40]. The cooking of green leafy vegetables resulted in considerably higher β-carotene retention (18–380%) after boiling them for 8 min, and retention was 2–3 times higher after stir-frying for 4 and 8 min, respectively [41]. These results demonstrate that cooking enhances the release of carotenoids by disrupting the food matrix. The differences in the retention of provitamin A in the different biofortified maize porridge samples of this study may be attributed to differences in provitamin A composition, with different provitamin A molecules presumably having different heat sensitivities.

### 3.3. Sensory Quality of the Porridges

**Figure 2:**
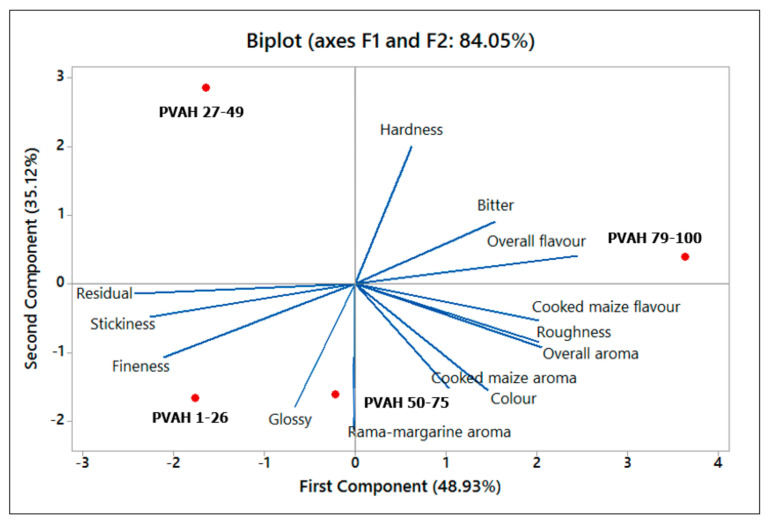
Biplot of principal component PC1 versus PC2 loadings for four provitamin A-biofortified maize stiff porridges. PVAH = Provitamin A hybrid.

The sensory attributes of the biofortified maize porridges, as described by the trained panel members, are presented in Table 4. The biofortified maize porridges were described as ‘sticky’ and ‘fine’, with intense ‘cooked maize’ flavour and aroma, compared to the representative white maize porridge in which these sensory attributes were either absent or insignificant. A ‘*Rama*-margarine’ aroma, bitter aftertaste, and residual grain were also perceived at much higher intensities than in the representative white maize porridge, where these sensory attributes were also either absent or insignificant. Table 4 indicates that, generally, the biofortified maize porridges had similar intensities of the observed characteristic sensory attributes. However, by principal component analysis (PCA), the biofortified maize porridges were separated based on these characteristic sensory attributes (Figure 2). The representative white maize porridge was not included in the PCA because it had insignificant intensities of the sensory attributes uniquely found in the biofortified maize porridges, i.e., bitter aftertaste, ‘*Rama*-margarine’ aroma, and residual grain. PC1 and PC2 explained 48.9% and 35.1% of the total variance, respectively. In PC1, the porridge made with PVAH 79-100 was separated from PVAH 1–26 porridge. The PVAH 79–100 porridge was characterized by a cooked maize flavour, roughness, overall aroma, ‘cooked maize’ aroma, and colour, whilst the PVAH 1–26 porridge was described as having an intense residual grain, stickiness, and fineness. In PC 2, the porridge made with PVAH 27–49 was separated from the PVAH 50–75 porridge. The porridge of the variety PVAH 27–49 was characterized by an intense hardness, whilst the PVAH 50–75 porridge was associated with a glossy appearance and ‘*Rama*-margarine’ aroma. Hardness was negatively correlated to glossiness and ‘*Rama*-margarine’ aroma (*p* < 0.05). The South African margarine brand ‘*Rama’* is yellowish in colour; however, carotenoid pigments may also be partly responsible for the observed colour. It is likely the carotenoid pigments in the provitamin A-biofortified maize porridges also contributed to the ‘*Rama*-margarine’ flavour detected in the porridges of this study. Fineness, stickiness, and residual grain may be linked to particle size of the meal. Carotenoid pigments may also have contributed to the stickiness of the porridges. Bitterness was probably caused by phenolic compounds in the maize. Cereal grains are known to contain phenolic compounds, which influences the taste of cereal-based foods [42]. The descriptive test panellists of this study commented that the two attributes ‘bitter aftertaste’ and ‘stickiness’ were not typical sensory properties of stiff white maize porridge. Bitter aftertaste and stickiness are therefore likely to negatively affect the sensory acceptability of provitamin A maize porridges compared to white porridge as discussed further below.

### 3.4. Sensory Acceptability of the Porridges

The acceptability of the porridges to the consumer panel is shown in Table 5. The sensory acceptability of the biofortified maize porridges was the same in terms of all the attributes evaluated. There was no significant difference between the sensory acceptability of the biofortified porridges and the white porridge with respect to all the attributes evaluated, including overall acceptability. However, numerically, the mean ratings for texture, taste, and overall acceptability of the biofortified porridges were generally lower than that of the white porridge. Overall, the biofortified maize porridges were rated ‘good’ (overall acceptability rated above 4). The lower ratings for the taste and texture of the biofortified porridges, relative to the white maize porridge, may be attributed to the bitter aftertaste and stickiness, which the descriptive panel reported. In addition, the bitter aftertaste and stickiness, which were described by the descriptive sensory panel (Table 4), could have contributed to the lower acceptability of the biofortified maize porridges relative to the white maize porridge. In a previous South African study on consumer acceptance of yellow maize meal, bitterness was also reported, and it was suspected to negatively affect the acceptability of the yellow maize meal [43]. Therefore, it appears that there is a need to reduce the bitter aftertaste and stickiness of provitamin A-biofortified maize. This could be achieved through recipe development.

### 3.5. Cluster Analysis of the Porridges

Consumers were divided into three clusters based on overall liking of the biofortified porridges (Table 6). Cluster 1 comprised of 38.3% of the consumer panel; this cluster of consumers neither liked nor disliked the biofortified maize porridges. Cluster 1 was dominated by female consumers (60.9%), with almost 50% of the consumers in this cluster having an age range of 41–60 years. The two age groups 20–40 years and 61–80 years had an equal proportion of consumers. Cluster 2 made up 33.3% of the consumer panel, and the consumers in this cluster liked the biofortified maize porridges. About 90% of the consumers in this cluster were female, and approximately 55% of the consumers were aged between 41 and 60 years, inclusively, whilst the other two age groups had a similar proportion of consumers. Cluster 3 represented the smallest consumer group (28.3% of the consumer panel). Consumers in this cluster disliked the biofortified maize porridges. Similar to the other two clusters, this cluster was dominated by females (58.8%). Approximately 58.8% of these consumers had an age range of 20–40 years, whilst 5.9% were aged 41–60 years and 35.3% had ages ranging from 61–80 years. Although the consumer panel rated the overall acceptability of the biofortified maize porridges to be as good as the white porridge, segmentation of the consumer panel showed there were differences in the acceptability of the biofortified maize porridges across consumer clusters. Overall, only 33.3% of the consumer sample liked the biofortified maize porridges (Table 6). The Chi-square test showed that in all three consumer clusters, consumer’s liking of the porridges was not correlated with gender, but was significantly correlated with consumer age (*p* < 0.05) (Table 6). Consumers in the middle age range (41–60 years) either liked the biofortified maize porridges (55% of the consumers in cluster 2), or were neutral (47.8% of the consumers in cluster 1). The highest proportion of consumers who disliked the biofortified maize porridges (58.8% of the consumers in cluster 3) were in the youngest age group (20–40 years of age). As reviewed earlier, Pillay et al. [10] also found consumer age influenced the acceptability of provitamin A-biofortified food products. However, Pillay et al. [10] found that it was the younger consumers (pre-school and younger school children) who liked the biofortified maize products more than the older consumers. Despite this seeming contradiction of age preferences, the findings of Pillay et al. [10] cannot be compared with the findings of this study, because their study included children whereas the current study did not. Amongst the three age groups in this study, the 20–40 years age group is comprised of the most sensory sensitive, socio-economically active, easily-influenced, and discriminating individuals. It is likely that their dislike of the biofortified maize porridges was due to a combination of these factors, for example, they are likely to have had a higher sensitivity to the undesirable sensory properties of the biofortified maize porridges than consumers of the other age groups. They are also more likely to have heard about the negative stigma attached to yellow maize and influenced each other to dislike it. As previously mentioned, the authors found that consumer gender was not associated with the acceptance of the biofortified maize foods.

Although there are limitations to the findings of this study, they suggest that interventions aimed at increasing consumer acceptance of provitamin A-biofortified maize, e.g., nutrition education, should primarily target consumers aged 20–40 years old. The age group of 41–60 years, where 55% of the consumers liked the biofortified maize porridges, could potentially assist with advocating for the consumption of biofortified maize.

## 4. Conclusions

Except for colour, the physical properties of provitamin A-biofortified maize were found to be similar to those of white maize and hence it can be processed, e.g., milling, in the same manner as white maize. There is substantial retention of provitamin A (91% to 123%) in the stiff porridges, suggesting that stiff porridge is a suitable food type for delivering provitamin A to targeted consumers. The provitamin A-biofortified maize stiff porridge can be described as ‘sticky’ (5.2–5.7), ‘fine’, with high intensity residual grain (2.7–2.9), a slightly bitter aftertaste (1.7–1.8) with cooked ‘maize flavour’ (4.9–5.1) and aroma (4.9–5.1) compared to the representative white maize porridge, in which these sensory attributes were either absent or insignificant. Some of these attributes, e.g., bitter aftertaste and stickiness, may have contributed to the low acceptance (4.5–4.6) of provitamin A-biofortified maize than representative white maize porridge (4.7). The results indicate that consumer age is a contributing factor to the acceptance of provitamin A-biofortified maize; it seems that when addressing the challenge of the low acceptance of the biofortified maize, it would be necessary to use different strategies on consumers of different age groups. Overall, the findings indicate that there is a need to improve the acceptance of provitamin A-biofortified maize, which can be achieved through recipe development and nutrition education.

## Figures and Tables

**Table 1:** Descriptors developed by the trained sensory evaluation panel for the profiling of the provitamin A-biofortified maize stiff porridges and the representative white porridge.

Attribute		Definition	References	Rating Scale
**Appearance**				
	Colour	Degree of colour intensity ranging from yellow to dark yellow.	Rama (rate 2) Deep yellow cheese	0 = light yellow 10 = deep yellow
	Glossy	Degree of glossiness (shiny) of porridge ranging from opaque (not glossy) to very shiny.	Rama margarine (South African Brand) Ultramel custard	0 = opaque 10 = shiny
	Roughness	The degree of roughness as seen on the surface of the porridge.	Ultramel custard Coarse white maize porridge (25% solid).	0 = not rough 10 = very rough
**Aroma**				
	Overall aroma	The overall aroma intensity of maize porridge		0 = not intense 10 = very intense
	Cooked maize aroma	The intensity of cooked maize aroma in the porridge.	Stiff coarse yellow maize porridge (25% solid)	0 = not perceived 10 = strongly perceived aroma
	*Rama*-margarine aroma	The intensity of *Rama*-margarine aroma in the porridge.	Cooked soft yellow maize porridge with Rama margarine (South African Brand).	0 = not perceived 10 = strongly perceived
**Texture**				
	Stickiness	The degree to which the porridge adhered to fingers.	Thin porridge (10% solid). White wheat dough	0 = not sticky 10 = very sticky
	Hardness	The force required to compress the porridge.	Thin cooked porridge (10% solid). Cooked coarse maize meal (33%)	0 = not hard 10 = very hard
	Fineness	The degree of fineness of granules felt in the mouth.	Coarse white maize meal (25%) Corn starch (25% solid)	0 = not fine 10 = very fine
**Flavour**				
	Overall flavour	The overall flavour intensity of maize porridge.		0 = not intense 10 = very intense
	Cooked maize flavour	The intensity of cooked maize flavour in the porridge.	25% cooked yellow maize porridge	0 = bland 10 = strong cooked maize flavour
**Aftertaste**				
	Bitter	The bitter sensation after swallowing the porridge.	Cold instant coffee solution (30% solid)	0 = Not intense 10 = very intense
	Residual grain	The extent to which the particles are felt in the mouth after swallowing.	Cooked coarse sorghum flour	0 = none 10 = a lot

**Table 2:** Physical properties of provitamin A-biofortified and representative white maize grains.

Variety	^d^ Colour (Hunter Values)			^dλ^ Grain Texture	^λ^ Grain Density	
	*L*	*a*	*b*	MI	^d^ TKW (g)	HLM (kg/hL)
RWM	46.3 ^b^ ± 1.1	6.3 ^a^ ± 1.1	24.6 ^a^ ± 2.6	93.5 ^c^ ± 1.8	257.1 ^a^ ± 5.4	78.5 ^a^
PVAH-79–100	37.2 ^a^ ± 1.3	16.6 ^b^ ± 3.0	28.1 ^ab^ ± 2.5	69.9 ^b^ ± 1 0.6	206.5 ^b^ ± 0.8	80.1 ^a^
PVAH-27–49	39.0 ^a^ ± 3.2	17.2 ^b^ ± 2.4	29.2 ^ab^ ± 2.5	79.0 ^a^ ± 1.6	234.4 ^c^ ± 0.2	81.5 ^b^
PVAH-50–75	39.0 ^a^ ± 1.9	17.0 ^b^ ± 0.3	28.7 ^ab^ ± 1.1	73.3 ^ab^ ± 2.1	227.7 ^d^ ± 0.0	78.8 ^a^
PVAH-1–26	36.6 ^a^ ± 0.7	18.9 ^b^ ± 2.3	31.0 ^bc^ ± 3.6	80.7 ^a^ ± 1.2	267.3 ^e^ ± 0.6	81.6 ^b^

^d^ Means ± Standard deviation. Mean values followed by different superscript letters in the same column are significantly different at *p* < 0.05 (LSD). Hunter values: *L* = black (0) to white (100); *a* = red (+) to green (-); *b* = yellow (−) to blue (+). MI = milling index; TKW = thousand kernel weight; HLM = hectolitre mass; PVAH = provitamin A hybrid; RWM = representative white maize. ^λ^ These parameters were measured on a dry matter basis.

**Table 3:** Provitamin A retention in provitamin A-biofortified maize stiff porridges and the representative white maize porridge.

Variety	Provitamin A Content (µg/g DW)		ProvA Retention (%)
	Maize Flour	Stiff Porridge	
RWM	n.d	n.d	n.d
PVAH1–26	2.58 ^a^ ± 0.06	3.18 ^a^ ± 0.01	123.30 ^a^ ± 2.93
PVAH27–49	2.47 ^ab^ ± 0.12	2.60 ^b^ ± 0.01	105.30 ^b^ ± 5.27
PVAH50–75	2.46 ^a^ ± 0.07	2.24 ^c^ ± 0.06	91.39 ^c^ ± 4.23
PVAH79–100	2.40 ^b^ ± 0.08	2.60 ^b^ ± 0.01	108.41 ^b^ ± 3.82

Means ± SD. Mean values followed by different letters in a column are significantly different *p* < 0.05 (LSD). PVAH= provitamin A hybrid; n.d = not detected; DW = dry weight; ProvA = provitamin A; RWM = representative white maize.

**Table 4:** Sensory attributes of the provitamin A-biofortified maize stiff porridges and representative white maize porridge.

Attribute	Maize Stiff Porridge Samples				
	RWM	PVAH 79–100	PVAH 27–49	PVAH 1–26	PVAH 50–75
Colour	0.0 ^a^ ± 0.0	4.9 ^b^ ± 1.0	4.0 ^a^ ± 0.8	4.6 ^ab^ ± 1.0	4.8 ^b^ ± 1.0
Glossy	3.1 ^a^ ± 0.9	3.6 ^ab^ ± 0.9	3.6 ^ab^ ± 0.8	4.0 ^b^ ± 0.9	3.8 ^b^ ± 1.1
Roughness	1.8 ^a^ ± 0.9	4.1 ^b^ ± 0.8	3.8 ^b^ ± 0.7	3.9 ^b^ ± 0.7	4.0 ^b^ ± 0.8
Overall aroma	1.5 ^a^ ± 0.8	4.4 ^b^ ± 1.3	4.3 ^b^ ± 1.3	4.4 ^b^ ± 1.2	4.3 ^b^ ± 1.2
Cooked maize aroma	1.4 ^a^ ± 0.9	5.1 ^b^ ± 1.9	4.9 ^b^ ± 2.0	5.0 ^b^ ± 1.9	5.1 ^b^ ± 1.9
*Rama*-margarine aroma	0.2 ^a^ ± 0.1	3.3 ^b^ ± 1.5	3.2 ^b^ ± 1.5	3.4 ^b^ ± 1.7	3.4 ^b^ ± 1.6
Stickiness	0.3 ^a^ ± 0.1	5.2 ^b^ ± 1.5	5.6 ^b^ ± 1.4	5.7 ^b^ ± 1.6	5.6 ^b^ ± 1.3
Hardness	1.4 ^a^ ± 0.8	4.1 ^b^ ± 1.4	4.2 ^b^ ± 1.5	3.8 ^b^ ± 1.3	3.9 ^b^ ± 1.4
Fineness	2.1 ^a^ ± 0.6	4.7 ^b^ ± 1.3	5.0 ^b^ ± 1.4	5.2 ^b^ ± 1.5	5.2 ^b^ ± 1.4
Overall flavour	1.2 ^a^ ± 1.0	4.0 ^b^ ± 1.9	3.7 ^b^ ± 1.6	3.7 ^b^ ± 1.6	3.5 ^b^ ± 1.6
Cooked maize flavour	1.1 ^a^ ± 0.2	5.1 ^b^ ± 1.9	4.9 ^b^ ± 1.9	5.1 ^b^ ± 2.0	4.9 ^b^ ± 2.0
Bitter	0.0 ^a^ ± 0.0	1.8 ^b^ ± 1.3	1.8 ^b^ ± 1.4	1.8 ^b^ ± 1.2	1.7 ^b^ ± 1.2
Residual	0.0 ^a^ ± 0.0	2.7 ^b^ ± 0.9	2.9 ^b^ ± 1.0	2.8 ^b^ ± 1.0	2.9 ^b^ ± 0.9

Means ± SD (*n* = 10). Mean values followed by different superscript letters in the same row are significantly different (*p* < 0.05). PVAH = Provitamin A hybrid; RWM = representative white maize.

**Table 5:** Sensory acceptability of provitamin A-biofortified maize stiff porridges.

Sensory Attribute	Provitamin A-Biofortified Maize Stiff Porridges				
	RWM	PVAH 27–49	PVAH 1–26	PVAH 50–75	PVAH 79–100
Colour	4.6 ^a^ ± 0.6	4.6 ^a^ ± 0.7	4.6 ^a^ ± 0.6	4.6 ^a^ ± 0.5	4.5 ^a^ ± 0.9
Texture	4.6 ^a^ ± 0.7	4.5 ^a^ ± 0.9	4.4 ^a^ ± 0.9	4.6 ^a^ ± 0.6	4.3 ^a^± 1.0
Taste	4.7 ^a^ ± 0.6	4.5 ^a^ ± 0.8	4.4 ^a^ ± 0.9	4.5 ^a^ ± 0.7	4.4 ^a^ ± 0.9
Aroma	4.6 ^ab^ ± 0.7	4.6 ^ab^ ± 0.7	4.4 ^a^ ± 1.0	4.7 ^b^ ± 0.5	4.5 ^ab^ ± 0.9
Overall acceptability	4.7 ^a^ ± 0.6	4.5 ^a^ ± 0.8	4.5 ^a^ ± 0.8	4.6 ^a^ ± 0.5	4.6 ^a^ ± 0.7

Means ± SD (n = 60). LSD (*p* < 0.05). Mean values not followed by different superscript letters are not significantly different. PVAH = Provitamin A hybrid; RWM = representative white maize variety. Five-point hedonic scale ranged from 1 to 5 (1 = “dislike extremely”, 2 = dislike moderately, 3 = neither like nor dislike, 4 = like moderately, 5 = “rather than”).

**Table 6:** Relationship between consumer segments, gender, and age.

Cluster	Cluster Description	Consumer	Gender		* *p*-Values	Age			* *p*-Values
		N, (%)	Male	Female		20–40	41–60	61–80	
1	Neither liked nor disliked	23 (38.3)	39.1	60.9	0.6	26.1	47.8	26.1	0.04
2	Liked	20 (33.3)	10.0	90.0		25.0	55.0	20.0	
3	Disliked	17 (28.3)	41.2	58.8		58.8	5.9	35.3	

N = 60. * *p*-values generated using Chi-square test.
